# Label-Free Electrochemical Sensor for Rapid Bacterial Pathogen Detection Using Vancomycin-Modified Highly Branched Polymers

**DOI:** 10.3390/s21051872

**Published:** 2021-03-08

**Authors:** Holger Schulze, Harry Wilson, Ines Cara, Steven Carter, Edward N. Dyson, Ravikrishnan Elangovan, Stephen Rimmer, Till T. Bachmann

**Affiliations:** 1 Infection Medicine, Edinburgh Medical School, College of Medicine and Veterinary Medicine, The University of Edinburgh, Chancellor’s Building, 49 Little France Crescent, Edinburgh EH16 4SB, UK; holger.schulze@ed.ac.uk (H.S.); harrywilson412@gmail.com (H.W.); ines.cara@grenoble-inp.org (I.C.); 2 Polymer and Biomaterials Chemistry Laboratories, School of Chemistry and Biosciences, University of Bradford, Bradford BD7 1DP, UK; S.R.Carter@bradford.ac.uk (S.C.); E.N.Dyson@bradford.ac.uk (E.N.D.); S.Rimmer@bradford.ac.uk (S.R.); 3 Department of Biochemical Engineering and Biotechnology, Indian Institute of Technology Delhi, New Delhi 110016, India; elangovan@iitd.ac.in

**Keywords:** electrochemical impedance spectroscopy (EIS), highly branched polymers, bacteria pathogen detection, label-free, point of care diagnostics, AMR

## Abstract

Rapid point of care tests for bacterial infection diagnosis are of great importance to reduce the misuse of antibiotics and burden of antimicrobial resistance. Here, we have successfully combined a new class of non-biological binder molecules with electrochemical impedance spectroscopy (EIS)-based sensor detection for direct, label-free detection of Gram-positive bacteria making use of the specific coil-to-globule conformation change of the vancomycin-modified highly branched polymers immobilized on the surface of gold screen-printed electrodes upon binding to Gram-positive bacteria. *Staphylococcus carnosus* was detected after just 20 min incubation of the sample solution with the polymer-functionalized electrodes. The polymer conformation change was quantified with two simple 1 min EIS tests before and after incubation with the sample. Tests revealed a concentration dependent signal change within an OD_600_ range of *Staphylococcus carnosus* from 0.002 to 0.1 and a clear discrimination between Gram-positive *Staphylococcus carnosus* and Gram-negative *Escherichia coli* bacteria. This exhibits a clear advancement in terms of simplified test complexity compared to existing bacteria detection tests. In addition, the polymer-functionalized electrodes showed good storage and operational stability.

## 1. Introduction

Infectious diseases and their growing resistance to antimicrobial drugs place an increasingly large burden on global public health, particularly in developing countries where diagnostic and specific treatment options are often lacking [1,2]. Conventional laboratory-based diagnosis techniques have long processing times and require specialist equipment. As a result, antimicrobial therapy is often started based on empirical evidence, without definitive diagnoses, leading to inappropriate overuse of broad-spectrum antibiotics and contributing to the rise in antimicrobial resistance (AMR) [3]. Rapid, specific, point-of-care tests to detect bacteria have huge importance for One Health. The DOSA project (DOSA-Diagnostics for One Health and User Driven Solutions for AMR) aims to develop diagnostic solutions which meet the user needs in terms of simplicity of use and rapid turnaround. The presented study is a prime example how the combination of a novel bacterial binding polymer and the highly sensitive electrochemical impedance spectroscopy can meet this demand.

Electrochemical impedance spectroscopy (EIS) is a label-free detection method which has been applied for various different types of targets ranging from entire bacteria detection with immobilized antibodies, over nucleic acid targets to proteins and small molecules [4,5,6,7,8,9,10,11,12,13,14,15,16,17,18]. We have previously developed in the Bachmann group EIS biosensors for the detection of the *mecA* gene and genomic DNA of methicillin-resistant *S. aureus* (MRSA), the New Delhi Metallo-beta-lactamase (NDM) carbapenem-resistance gene and for bacterial 16S ribosomal RNA for bacterial species identification [19,20,21,22].

Highly branched poly(N-*iso*propyl acrylamide) (HB-PNIPAM-Van) with vancomycin modified ends have been synthesized by the Rimmer group [23,24,25,26]. These polymers have a highly branched structure and undergo conformational change between an open coil form and a globular form. This has been shown to be induced by changes in temperature and interaction with bacteria [24,26]. Vancomycin is an antibiotic, which binds to the D-Ala-D-Ala peptide of peptidoglycan cell walls, making it specific to Gram-positive bacteria.

In this paper, we combine vancomycin-modified highly branched polymers as a new class of non-biological binder molecules for bacteria with the advantage of label-free EIS detection for direct, rapid label- and amplification-free bacteria detection.

## 2. Materials and Methods

### 2.1. Highly Branched Poly(N-isopropyl acrylamide) Polymers 

Highly branched poly(N-*iso*propyl acrylamide) polymers (HB-PNIPAMs) were prepared using our previously reported technique [25]. Full details are provided in Appendix A. Briefly, a polymer was prepared by self-condensing vinyl copolymerisation of NIPAM (25 molar equivalents) and 4-vinylbenzyl 1*H*-pyrrole-1-carbodithioate (1 molar equivalent). The polymerisation produced a branched material with pyrrole carbodithioate end groups. The end groups were modified by reaction with differing amounts of azobis(cyanovalearic acid) at 60 °C in dioxane. The resultant polymers with a mixture of carboxylic acid and pyrrole carbodithioate end groups were partially functionalised with vancomycin via the succinimide ester. Three polymers were produced which were defined in terms of two parameters: the percentage of branch points leading to a vancomycin group (f_van_) and the percentage of branch points leading to a pyrrole end group (f_py_). 

Polymer solutions (5g dm^−3^) were prepared using HB-PNIPAM (5mg) mixed in phosphate-buffered saline (PBS, 1mL) buffer (1× PBS solution (11.9 mM phosphates + 137 mM sodium chloride + 2.7 mM potassium chloride), pH 7.4) in an ice bath at 0–1 °C.

### 2.2. Electrode Functionalization

Screen-printed gold electrodes with a 1.6 mm in diameter gold working electrode, a silver (Ag) reference electrode and a gold counter electrode (DRP-223BT, Metrohm-Dropsens, Oviedo, Spain) stored under vacuum were used for all tests. Pre-treatment of the silver reference electrode by 30-s coverage with 6 µL 50 mM FeCl_3_ solution, to form an Ag/AgCl reference electrode. Then, washed with 5mL deionized water and dried under a stream of Argon. Electrodes were cleaned using cyclic voltammetry, under a 100 µL drop of 0.1 M sulfuric acid, daisy-chain connected to a potentiostat via connector boxes (Metrohm-Dropsens, Oviedo, Spain), using ten cycles between 0 and 1.6 V and three cycles between 0 and 1.3 V at 100 mV/s. Cleaned electrodes were then washed with 5mL deionized water and dried under a stream of Argon. Electrodes were functionalized by immobilization of HB-PNIPAM polymer on the surface of the gold working electrodes. This was achieved by incubation of working electrode with 4.5 µL of the relevant polymer solution in PBS (see Table 1), for 16–20 h in 70% humidity (humidification chamber created by saturated KCl solution). Functionalization was stopped by washing electrodes with 4 × 1 mL ice cold (0–1 °C) deionized water then incubating in cold (0–1 °C) deionized water for 10 min (40 mL in a 100 mL glass beaker to ensure electrode surface coverage). Functionalized electrodes were stored at 4 °C until and between measurements.

### 2.3. Bacterial Culture

A single colony of *Escherichia coli* (*E. coli*) *DH5α*, *Staphylococcus carnosus* (*S. carnosus*) ATCC 51365, or a negative control was mixed into 5mL of Luria-Bertani (LB) medium (10 g/L Bacto-tryptone, 5 g/L yeast extract, 10 g/L NaCl). Then, incubated overnight (16‒18 h) at 37 °C and 175 rpm in a shaking incubator (Infors HT, Bottmingen-Basel, Switzerland). The cultures were centrifuged at 3500 rpm and 4 °C for 5 min, the supernatant removed, and the pellet re-suspended in 5 mL PBS. This was repeated. The optical density (OD_600_) was measured, relative to a PBS blank, in a spectrophotometer, and used to prepare bacterial target solutions of a certain OD_600_.

### 2.4. EIS Measurements

All EIS measurements were performed with an EIS buffer containing 1 mM potassium Ferricyanide (K_3_[Fe(CN)_6_] (Sigma Aldrich 455946-25G) and 1 mM potassium Ferrocyanide (Sigma Aldrich 455989-25G) dissolved in 1× PBS pH 7.4 (11.9 mM phosphates + 137 mM sodium chloride + 2.7 mM potassium chloride). EIS measurements (and electrode cleaning) were conducted using Metrohm Autolab potentiostats PGSTAT128N (Utrecht, Netherlands) with integrated multiplexor module allowing 3-4 electrodes to be tested sequentially; running NOVA 2.1 software. EIS measurements were performed at open circuit potential with an amplitude of 10 mV rms using a frequency range between 100,000 Hz and 0.3 Hz (20 frequencies) in EIS buffer. Data were plotted as Nyquist and Bode plots and fitted using the Randles equivalent circuit model [R_s_([R_ct_W]C)] comprising of a solution resistance (R_s_), the charge transfer resistance (R_ct_), the double layer capacitance (C) and the Warburg impedance (W). Before each EIS measurement, electrodes, in the measurement cell, were washed with, then incubated in, EIS buffer for 5 min. All EIS measurements were carried out in closed measurement cells with a sample volume of 80 µL.

### 2.5. Temperature-Induced Polymer Conformation Change

EIS measurements were performed in closed measurement cells preventing evaporation with 80 µL EIS buffer added at incremental increases in temperature, using a Stuart SD160 Digital Hotplate (Keison Products, Chelmsford, UK) to raise the solution temperature within the measurement cell. This was repeated at room temperature (RT), 25 °C, 30 °C, 35 °C, 40 °C, 45 °C, and a final measurement, with the cell removed from the hotplate, after 15 min to return to room temperature (RT2). Finally, all electrodes were measured again at room temperature (RT3) one day after the original temperature increase measurements. These final measurements were to investigate the short- and long-term recovery of the polymers.

### 2.6. Bacteria Detection

Tests were performed on electrodes functionalized with the HB-PNIPAM-Van-C highly branched polymer, before and after incubation with *S. carnosus, E. coli* and PBS buffer as target solutions, respectively. The functionalized electrodes were incubated with the target solution within the measurement cell at 30 °C (hotplate set at 32 °C) or 35 °C (hotplate set at 37 °C) for 20 min or 2 h. Post-incubation, the target solution was removed, and the electrode/cell was washed with EIS buffer. EIS measurements were done before and after incubation with the target solutions to compare the change in the R_ct_ values from before to after target incubation (signal change ratio). Alternatively, after the baseline measurement, electrodes were removed from the measurement cell and incubated in a 15 mL reaction tube containing 3.5 mL of the relevant target solution (surface covered but not connectors) for 2 h at 21 °C, 30 °C or 35 °C in a thermomixer with a 15 mL tube adapter (Eppendorf, Hamburg, Germany). Electrodes were then washed in PBS and returned to measurement cells for the second measurement.

## 3. Results and Discussion

### 3.1. Polymer Characterisation

**Scheme 1:**
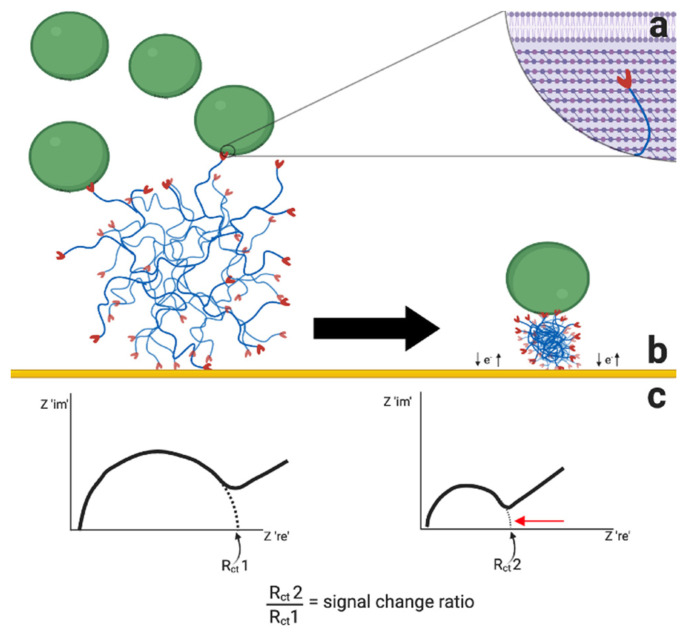
Test principle of the EIS-based sensor for detection of Gram-positive bacteria with vancomycin-modified HB-PNIMAM polymers immobilized on gold electrodes (**a**), which undergoes a coil-to-globule phase transition upon binding to Gram-positive bacteria (**b**) and thus causes a reduction in the R_ct_ value in the EIS Nyquist plot (**c**); the figure was created with BioRender.com (accessed on 1 February 2021).

Scheme 1 depicts the test principle of the new bacteria detecting EIS sensor with vancomycin-modified HB-PNIMAM polymers immobilized on the surface of gold electrodes, acting as the recognition element for specific detection of Gram-positive bacteria via binding of vancomycin to D-Ala-D-Ala in the cell wall of Gram-positive bacteria (a) [26]. This binding of the Gram-positive bacteria on the vancomycin-modified HB-PNIMAM polymers causes a coil-to-globule phase transition of the highly branched vancomycin-modified polymer (b). The condensed globule form of the polymer occupies less surface area on the electrode allowing greater redox exchange between the electrode surface and the redox active species in solution (Ferri/Ferrocyanide) (b). This results in a smaller charge transfer resistance (R_ct_) value, as shown by the change in shape of the Nyquist plot of the recorded EIS spectra (c).

**Figure 1:**
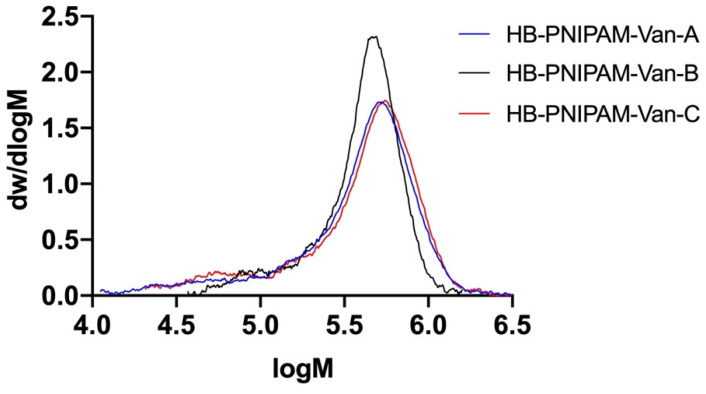
Molar mass distributions of the HB-PNIPAM-Van polymers obtained by size exclusion chromatography in methanol with refractive index and viscometric detection.

For the attachment of the vancomycin-modified HB-PNIPAM polymers, we have tested three different types of vancomycin-modified polymers with different ratios of vancomycin to pyrrole carbodithioate end groups (S = C(Z)S-R), the active agents of the reversible addition–fragmentation chain transfer (RAFT) reaction to form the highly branched polymers. The sulfur atoms in the pyrrole carbodithioate functional groups are capable of forming gold-sulfur bonds to attach the polymers onto the gold electrode surface. The molar mass averages of the polymers, the functionalities and the coil-to-globule lower critical transition temperatures are shown in Table 1 and full details of the synthesis are included in Appendix A. Figure 1 also shows the molar mass distributions obtained by size exclusion chromatography in methanol [27]. The molar mass distributions of the three highly branched vancomycin-modified polymers with different vancomycin to pyrrole carbodithioate end group ratios were similar and monomodal but with clear low molar mass tales, as can be seen. 

### 3.2. Electrode Functionalization with Vancomycin-Modified Highly Branched Polymers

**Figure 2:**
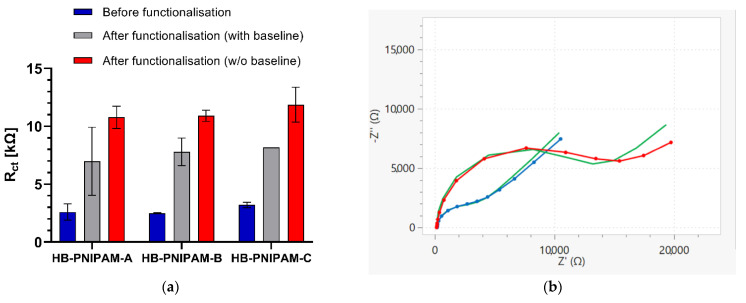
(**a**) Functionalization of gold screen-printed electrodes with three different types of vancomycin-modified highly branched polymers (HB-PNIPAM-A, B, C; see Table 1) shown by increased R_ct_ values after polymer immobilization on electrodes that were tested before and after (with baseline, grey bars) and only after polymer attachment (without baseline, red bars) compared to the R_ct_ values of bare, unmodified gold electrodes (blue bars), respectively; data are presented as mean ± standard error of the mean (SEM); n = 3. (**b**) EIS Nyquist plot of an unmodified, bare gold electrode (blue line + symbol) and an electrode tested after functionalization with the HB-PNIPAM-Van-C polymer without an initial baseline EIS measurement (red line + symbol); the green lines show the fitted data using the Randles equivalent circuit model.

**Figure 3:**
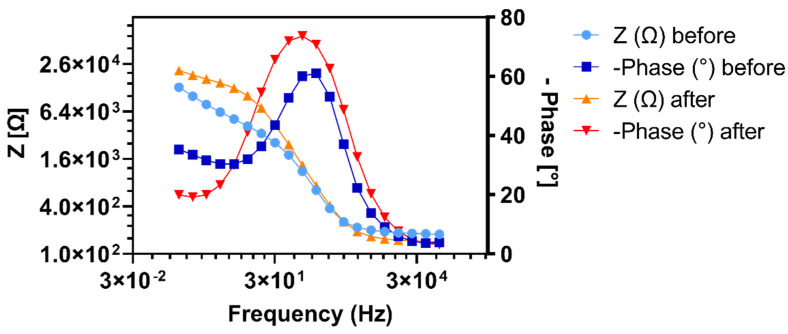
Bode plot EIS spectra of the same electrodes as shown in Figure 2b with the phase shift (dark blue) and absolute values of the impedance (light blue) of the unmodified, bare gold electrode plotted against the tested frequencies and the phase shift (red) and absolute values of the impedance (orange) of an electrode tested after functionalization with the HB-PNIPAM-Van-C polymer without an initial baseline EIS measurement.

Vancomycin-modified highly branched polymers were immobilized on gold screen-printed electrodes by incubating the surface of the gold working electrodes with a small volume of the polymer solution in PBS covering the working electrode area for 16–20 h in a humidification chamber. The successful attachment of the polymer onto the electrode surface was confirmed by EIS measurements before and after the polymer immobilization. As can be seen in Figure 2, the charge transfer resistance (R_ct_) value obtained from the Faradaic EIS measurement increased from a mean R_ct_ value of R_ct_ value of around 2.6 kΩ of the untreated, bare gold electrode to 10–15 kΩ after the polymer attachment. Figure 2b shows Nyquist plot EIS spectra, where the real part of the impedance Z’ on the a-axis is plotted against the imaginary part of the impedance –Z’’ on the y-axis, of such a bare gold electrode (blue) and of an electrode after functionalization with the highly branched vancomycin-modified polymer HB-PNIPAM-Van-C (red). This increase in the R_ct_ value, represented by the increase of the width of the semi-circle in the Nyquist plot after the attachment of the polymer onto the working electrode surface, is in agreement with previous findings and follows the theory that large compounds that are attached to the electrode surface reduce the electron transfer between the electroactive species in solution, here Ferri– and Ferrocyanide, and the electrode surface and thus increase the charge transfer resistance (R_ct_). The green lines in Figure 2b show the fitted data using the Randles equivalent circuit model [R_s_([R_ct_W]C_dl_)] comprising of a solution resistance (R_s_), the charge transfer resistance (R_ct_), the double layer capacitance C_dl_ and the Warburg impedance (W). Figure 3 shows the Bode plot EIS spectra of the same electrodes as shown in Figure 2b with the phase shifts and absolute values of the impedance plotted against the tested frequencies. Figure 2a shows the R_ct_ values, which were obtained from the data fitting with the Randles equivalent circuit, of bare, untreated gold electrodes (baseline; blue bars) and R_ct_ values after functionalization with polymers on electrodes which were tested before and after polymer immobilization (with baseline EIS measurements; grey bars) and electrodes that were only tested after the polymer immobilization (without baseline; red bars). Electrodes that were tested before and after the polymer immobilization showed slightly lower R_ct_ values after the polymer immobilization compared to the ones that were only tested after the polymer immobilization. The lower R_ct_ values on electrodes that were tested and thus exposed to EIS buffer before the polymer immobilization can be explained by a reduced cleanliness of the gold surface, which is required for the gold thiol bond formation in self-assembled monolayers.

Figure 2a shows that there was no significant difference in the amount of attached vancomycin-modified polymers between the polymers that contained different ratios of vancomycin to pyrrole carbodithioate functional end groups (HB-PNIPAM-Van-A, HB-PNIPAM-Van-B, HB-PNIPAM-Van-C). After modification of the end groups, first with 4,4’-azobis(4-cyanovaleric acid) (ACVA) and then by amidation with vancomycin, all three tested polymers still contained unreacted pyrrole carbodithioate end groups, as can be seen from the ^1^H-NMR data in Table 1. These residual pyrrole carbodithioate groups seem to be sufficient to form enough gold-sulfur bonds within the large polymer structure to enable a stable and lasting immobilization of the polymers on the gold electrode surface [28,29]. Besides the gold-sulfur bond formation, hydrophobic interactions between the polymer and the electrode surface will most likely also contribute to the polymer attachment.

**Figure 4:**
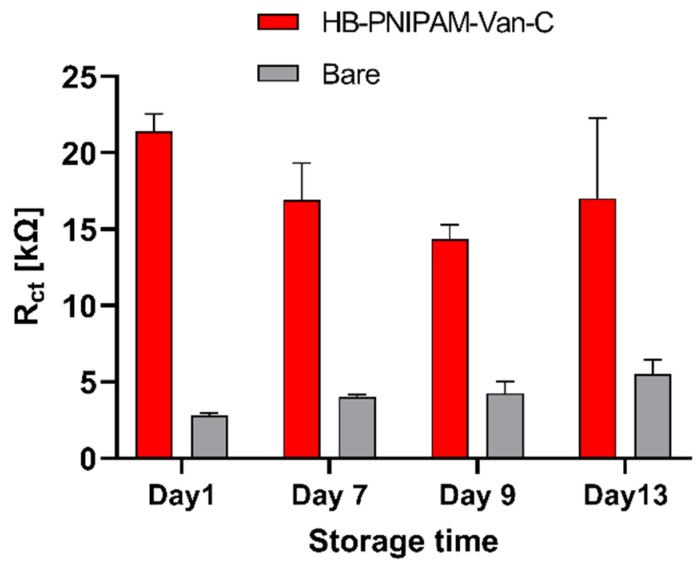
Storage stability of vancomycin-modified polymer HB-PNIPAM-Van-C functionalized electrodes demonstrated by stable R_ct_ values detected on a single set of polymer functionalized electrodes retested four times over 13 days (red bars); a set of bare, unmodified gold electrodes was re-tested over the same period of time for comparison (grey bars); data are presented as mean ± SEM; n = 3.

The operational and storage stability of the polymer functionalized electrodes was further investigated. Repeated tests of a set of electrodes functionalized with the vancomycin-modified highly branched polymer HB-PNIPAM-Van-C over a time period of 13 days with the electrodes stored dry in between at 4 °C and washed with ice-cold water before each new measurement showed an excellent storage and operational stability of these sensors, which is usually not achieved with antibody functionalized biosensors (Figure 4). This reveals a major advantage of this new type of non-biological binder molecules compared to existing biological recognition elements like for example antibodies or aptamers for protein and whole cell detection.

### 3.3. Temperature-Induced Conformation Change of Immobilized Highly Branched Vancomycin-Modified Polymers

**Figure 5:**
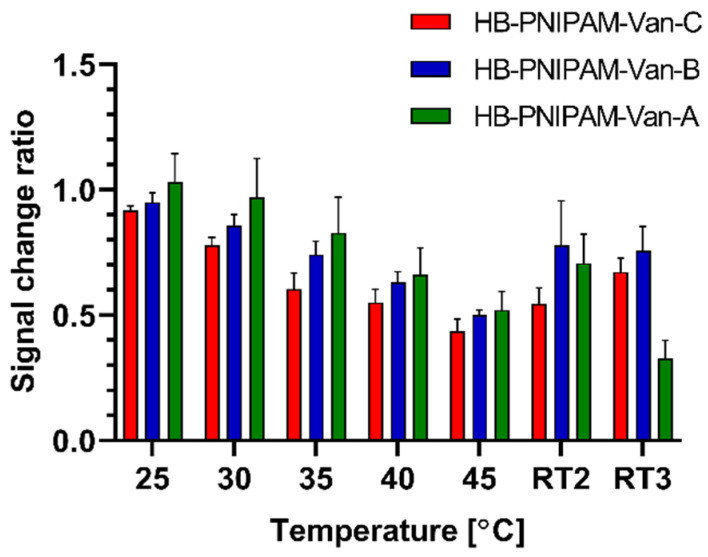
Temperature-induced R_ct_ signal change ratios of electrodes immobilized with three different vancomycin-modified highly branched polymers tested after incubation at different temperatures with EIS measurements; the mean R_ct_ values for each measurement are expressed as a ratio relative to the first RT measurement; data are presented as mean ± SEM; n = 4.

The effect of temperature on the behaviour of immobilized vancomycin-modified highly branched polymers was investigated using EIS sensors. Previously published data showed a temperature-induced coil-to-globule transition of highly branched polymers in solution [24]. Here, we test if the immobilized polymers follow the same open coil-to-globule transition upon temperature increase and the revers transition from the globule-to-coil form upon the reduction in the temperature back to room temperature (RT) of 21 °C. To do this, functionalized electrodes were subjected to incremental temperature increases, tested at different temperatures with EIS, and then measured again at room temperature (RT) to test their ability to recover from any changes. A temperature induced globule-to-coil transition would cause the polymer to occupy a reduced surface area on the electrode and therefore reduce the measured R_ct_ value. Overall, all tested electrodes with different functionalized polymers showed a downward trend of measured R_ct_ values with increasing temperature and partial recovery when returned to RT, as can be seen in Figure 5. These results are in agreement with the existing observations from the literature that temperature causes a coil-to-globule transition in PNIMAM polymers [30]. This occurs at a ‘lower critical solution temperature’ (LCST), thought to be above 32°C—this was verified for vancomycin-modified highly branched PNIMAN by Sarker et al. [24]. These results suggest a significant conformation change occurring between the 30 °C and 35 °C intervals.

### 3.4. Vancomycin-Modified Highly Branched Polymer Functionalised Electrode-Based EIS Detection of Bacteria

**Figure 6:**
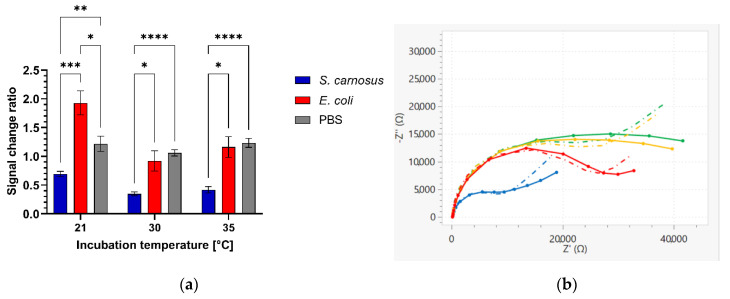
(**a**) R_ct_ signal change ratio after incubation of vancomycin-modified highly branched polymer HB-PNIPAM-Van-C functionalized electrodes with *S. carnosus* and *E. coli* suspensions in PBS (OD_600_ = 0.2) and with PBS buffer alone for 2 h in a 15 mL reaction tube with the electrodes covered with 3.5 mL bacteria solution at 21 °C (RT), 30 °C, 35 °C, respectively; results presented as a ratio of mean R_ct_ for an electrode after incubation with the target relative to the same value before incubation; data are presented as mean ± SEM; significance was determined using ANOVA multiple comparison tests; * *p* < 0.05, ** *p* < 0.01, *** *p* < 0.001, **** *p* < 0.0001; n = 6. (**b**) EIS Nyquist plots of vancomycin-modified highly branched polymer HB-PNIPAM-Van-C functionalized electrodes before and after incubation with *S. carnosus* (green: before; blue: after) and *E. coli* (orange: before; red: after) at 35 °C, respectively. The dash-dotted lines at the matched colours represent the fitted data based on the Randles equivalent circuit.

**Figure 7:**
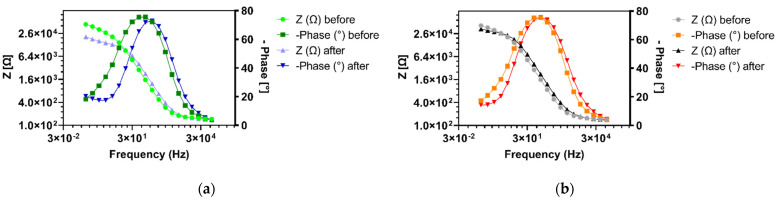
EIS Bode plots of vancomycin-modified highly branched polymer HB-PNIPAM-Van-C functionalized electrodes before and after incubation with *S. carnosus* (**a**) and *E. coli* (**b**) at 35 °C, respectively.

Finally, we have investigated the capability of the vancomycin-modified highly branched HB-PNIPAM-Van-C polymer sensors to specifically detect Gram-positive bacteria. *S. carnosus* was used as a non-pathogenic Gram-positive model analyte and the specificity for the detection of Gram-positive bacteria was demonstrated with tests with *E. coli* DH5α as a representative of a Gram-negative bacterium. Bacteria detection tests were performed at room temperature (21 °C), 30 °C, and 35 °C, respectively. The incubation with Gram-positive *S. carnosus* bacteria caused at all three incubation temperatures a reduction in the charge transfer resistance value (R_ct_) detected in the EIS measurement as depicted in Figure 6 as a signal change ratio smaller than one of the blue bars. Within which electrodes that were incubated with *S. carnosus* at elevated temperatures of 30 °C or 35 °C showed a larger reduction in the R_ct_ value after the 2 h incubation with the *S. carnosus* solutions compared to the room temperature incubation. There was now significant difference observed between 30 °C and 35 °C. Whereas, vancomycin polymer functionalized electrodes that were incubated with *E. coli* instead showed no reduction in the measured R_ct_ value after sample incubation. Figure 6b and Figure 7 show Nyquist plots and Bode plots of HB-PNIPAM-Van-C polymer functionalized electrodes before and after incubation with *S. carnosus* and *E. coli* at 35 °C, respectively. At all three tested incubation temperatures the signal change ratios after incubation with *S. carnosus* and *E. coli* were significantly different, thus demonstrating the capability of the vancomycin-modified highly branched polymer functionalized sensors to specifically detect Gram-positive bacteria. 

Different procedures for the incubation of bacteria with functionalized electrodes were tested. The functionalized electrodes were either incubated in the measurement cell, which was also used for the EIS measurements with a sample volume of 80 µL covering all three electrodes of the screen-printed electrode strip (working, counter, and reference electrodes) or with the screen-printed electrode strips inside a 15 mL reaction tube with the electrodes submerged in 3.5 mL bacteria solution. The temperature control during the bacteria incubation step was achieved with a hotplate in the case of the former measurement cell incubation with the measurement cells containing a heat conducting aluminum base and in the case of the reaction tube incubation with a thermomixer with a 15 mL tube adapter.

**Figure 8:**
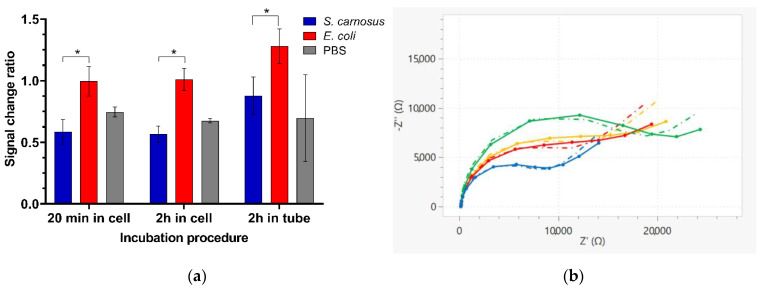
(**a**) R_ct_ signal change ratio after incubation of vancomycin-modified highly branched polymer HB-PNIPAM-Van-C functionalized electrodes with *S. carnosus* and *E. coli* suspensions in PBS (OD_600_ = 0.1) and with PBS buffer alone for 20 min or 2 h in the measurement cell with 80 µL sample volume or in a 15 mL reaction tube with the electrodes covered with 3.5 mL bacteria solution at 30 °C, respectively; results presented as a ratio of mean R_ct_ for an electrode after incubation with the target relative to the same value before incubation; data are presented as mean ± SEM; significance was determined using ANOVA multiple comparison tests; * *p* < 0.05; n = 4. (**b**) Nyquist plot EIS spectra of HB-PNIPAM-Van-C functionalized electrodes tested with *S. carnosus* (green: before incubation with *S. carnosus*; blue: after incubation) and *E. coli* (orange: before incubation with *E. coli*; red: after) (OD_600_ = 0.1, 2 h incubation in measurement cell). The dash-dotted lines at the matched colours represent the fitted data based on the Randles equivalent circuit.

**Figure 9:**
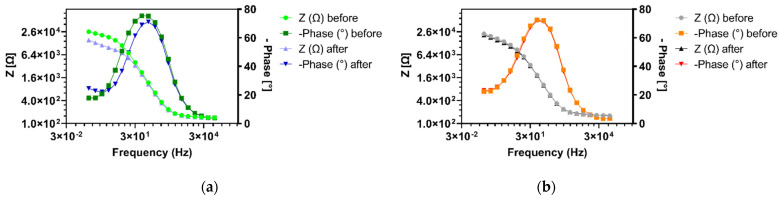
EIS Bode plots of vancomycin-modified highly branched polymer HB-PNIPAM-Van-C functionalized electrodes before and after incubation with *S. carnosus* (**a**) and *E. coli* (**b**) at 30 °C for 2 h in the measurement cell, respectively.

The *S. carnosus* binding induced coil-to-globule polymer conformation change of the immobilized HB-PNIPAM-Van-C was directly detected by a reduction in the R_ct_ value after bacteria incubation obtained from two rapid label-free EIS measurements before and after incubation of the functionalized electrodes with the bacteria solution. The measurement time to generate an EIS Nyquist plot and thus the Rct value is only one minute, making it a rapid and simple test with much reduced test complexity compared to other existing bacteria detection diagnostic tests [3,31,32]. For each tested procedure, the greatest reduction in R_ct_ signal was detected after incubation with *S. carnosus*, as can be seen in Figure 8. In contrast, the EIS signal remained basically unchanged after incubation with the Gram-negative *E. coli*, which does not bind to the vancomycin residues within the highly branched polymers and therefore not induce the coil to globule transition of the polymer (Figure 8). These data demonstrate the capability of the polymer sensor to discriminate between the two different types of bacteria and thus between Gram-positive and Gram-negative bacteria. The difference between obtained signal change after incubation with the two different types of bacteria was significant in each case (20 min, * *p* = 0.0222; 2 h in cell, * *p* = 0.0206; 2 h in tube, * *p* = 0.0367). Multiple comparisons did not reveal a significant difference between incubation procedures for the same treatment (*p* > 0.05). Figure 8b and Figure 9 show exemplar Nyquist and Bode plot EIS spectra of HB-PNIPAM-Van-C functionalized electrodes before and after 2 h incubation with *S. carnosus* and *E. coli* in the measurement cell, respectively. The observed EIS signal reduction after *S. carnosus* confirms the hypothesis that *S. carnosus* binding induces a conformational change of the polymer and is in line with existing literature showing Gram-positive bacteria causing a coil-to-globule transition in these polymers induced by the vancomycin binding to the D-Ala-D-Ala peptide of peptidoglycan cell walls of Gram-positive bacteria [24,26,33].

**Figure 10:**
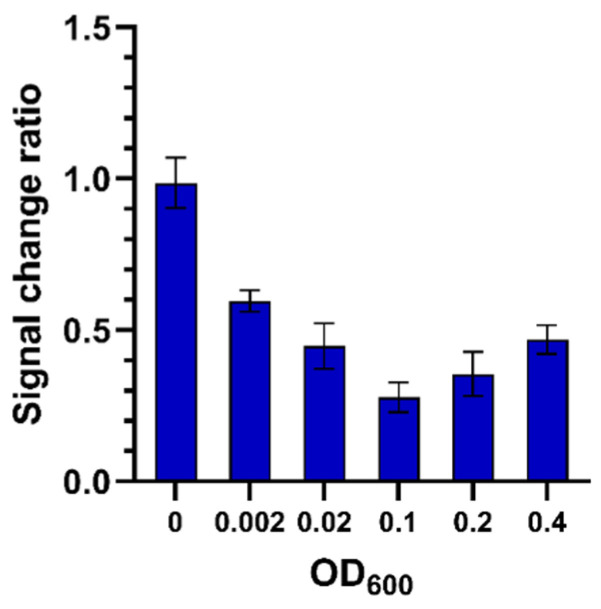
R_ct_ signal change ratio after 2 h incubation with different concentrations of *S. carnosus* Gram-positive bacteria (bacterial suspensions with an OD_600_ of 0, 0.002, 0.02, 0.1, 0.2, 0.4 at 35 °C, respectively); data are presented as mean ± SEM; n = 4.

Figure 10 shows a concentration dependent signal change when incubating the vancomycin highly branched polymer-functionalized electrodes with different concentrations of *S. carnosus* bacteria at 35 °C. The *S. carnosus* binding induced signal reduction increased with increasing *S. carnosus* concentrations up to an optical density (OD_600_) of 0.1. There was no further signal reduction observed from higher bacteria concentrations.

As a next step, we are planning to combine the Gram-positive bacteria specific vancomycin-modified highly branched polymers used in this study with highly branched poly(N-*iso*propyl acrylamide) polymers with polymyxin end groups. These polymyxin-modified polymers are specifically responsive to Gram-negative bacteria, such as *Pseudomonas aeruginosa*, as previously shown by Sarker et al. [23]. We plan to combine vancomycin- and polymyxin-modified highly branched polymers functionalized on separate working electrodes in an electrode array with two or more working electrodes with a combined reference and working electrode to detect both Gram-positive and Gram-negative bacteria in a single test. 

Such a rapid test that can distinguish between Gram-positive and Gram-negative bacteria aims to enhance the diagnostic pathway and thus patient care by aiding clinicians to choose the right antibiotic to treat bacterial infections such as urinary tract infections or blood stream infections where empirical antibiotic therapy is still the current practice in many healthcare settings [34,35]. This has the potential to reduce inappropriate antibiotic therapy, which will also reduce the rise of resistant bacteria and has therefore great potential to reduce the overall burden of AMR.

## 4. Conclusions

We have developed a new type of electrochemical impedance spectroscopy sensor for direct, label-free detection of bacteria already after 20 min incubation with a simple 1 min EIS test before and after incubation of the polymer functionalized sensor with the bacteria, respectively. This is to the best of our knowledge the first example for the use of highly branched poly(N-*iso*propyl acrylamide) polymers as non-biological recognition element in an electrochemical sensor. The functionalized polymer electrodes showed excellent dry storage and operational stability in repeated tests over a time period of 13 days. The results shown here provide proof of concept for the use of EIS with HB-PNIPAM polymers for distinguishing between Gram-positive and Gram-negative bacteria. This is an important step towards the development of an EIS-based rapid point-of-care diagnostic test for bacterial infection diagnosis to optimize patient treatment and reduce the miss-use of antibiotics.

## Figures and Tables

**Table 1:** Characterization data for HB-PNIPAM-Van polymer materials. ^a^ Molar masses obtained using SEC (methanol, 3 × 30 cm PolarGel columns (Agilent PLC) and using RI and viscometry). ^b^ F_van_ = percentage of branch points terminating in vancomycin end groups and ^c^ F_py_ percentage of branch points terminating in pyrrole carbodithioate end groups (^1^H-NMR)). ^d^ Lower critical solution temperature (LCST) obtained by calorimetry.

	$\bar{M_{n}}$ [kg mol^−1^] ^a^	$\bar{M_{w}}$ [kg mol^−1^] ^a^	$\bar{M_{z}}$ [kg mol^−1^] ^a^	F_van_ [%] ^b^	F_py_ [%] ^c^	LCST ^d^ [°C]
HB-PNIPAM-Van-A	205.5	490	704	1.51	3.42	29.9
HB-PNIPAM-Van-B	304.9	445	550	1.40	5.06	27.3
HB-PNIPAM-Van-C	230.0	504	709	0.42	7.01	24.1

## Appendix A

**Synthesis of highly branched poly(N-*iso*propyl acrylamide) polymers (HB-PNIPAM) with N-pyrrole carbodithioate end groups**

N-*Iso*propylacylamide (10.0 g, 88.37 mmol) and 4-vinylbenzylpyrrolecarbodithioate (0.9170 g, 3.535 mmol) were added to a 2-necked RB flask fitted with water condenser/N_2_ bubbler then 4,4’-azobis(4-cyanovaleric acid) (0.9908 g, 3.535 mmol) was added. The reactants were dissolved by the addition of dioxane (62.5 mL) under an N_2_ atmosphere then the contents of the flask were degassed by bubbling N_2_ through the solution for 30 min. The reaction mixture was heated at 60 °C for 48 h under a continual flow of N_2_. The solution was allowed to cool to room temperature then re-precipitated dropwise into rapidly stirring diethyl ether (1200 mL). Upon the settling of the solids, the ether was decanted off each sample and the solids washed with two consecutive portions (ca. 50 mL) of ether. The solid was dried *in vaccuo* at 35 °C (18 h) then re-dissolved in 1:1 *v/v* dioxane/ethanol (40 mL) and further re-precipitated into rapidly stirring diethyl ether (1200 mL). The solid was isolated as previously to give, following drying *in vaccuo*, 8.574 g (72%) of a white-yellow coloured solid.

The reactions were carried out on Carousel reactor (Radleys) in 100 mL 2N-RB flasks; water circulator; condenser at 10 °C. Flow of N_2_ through central inlet of carousel reactor; outlet needles in septa cap in each vessel head; Ultrafiltration was carried out using a Millipore ultrafiltration cell (300 mL) and 10,000 MWCO cellulose filters.

**Syntheses of HB-PNIPAM with pyrrole carbodithioate and carboxylic acid end groups (one portion ACVA)**

(a) The HB-PNIPAM with pyrrole dithioate end groups (1.00 g) was dissolved with stirring in dimethylformamide (DMF; 15 mL) in a 100 mL flask on a carousel reactor over 20 min. The reagent 4,4’-azobis(4-cyanovaleric acid) (ACVA, 1.8156 g, 6.478 mmol) was added to the solution under a N_2_ atmosphere and stirred at room temperature until full dissolved. The solution was degassed with N_2_ for 30 min with stirring then heated at 60 °C for 18 h. The solution was allowed to cool to room temperature then re-precipitated into rapidly stirring diethyl ether (300 L, the ether decanted off and the solids dried in a vacuum oven at 35 °C for 18 h. The solids were dissolved in ethanol (50 mL) then the solution subjected to ultrafiltration with ethanol (3 × 300 mL) to eventually give a polymer solution (ca. 30 mL), which following rotary evaporation gave 0.8040 g (80%) of an orange-yellow glassy solid.
(b) The procedure in (a) was repeated but before precipitation a second portion of ACVA (1.8156 g, 6.478 mmol) was added to the solution and stirring continued at 60 °C for a further 18 h.
(c) The procedure in (a) was repeated but before precipitation a second and third portion of ACVA (1.8156 g, 6.478 mmol) was added to the solution and stirring continued at 60 °C for a further 18 h.

**Synthesis of HB-PNIPAM-Van**

The HB-PNIPAM from procedure (a), (b) or (c) (0.700 g) was dissolved in DMF (8.0 mL) and stirred under a N_2_ atmosphere. A mixture of N-hydroxysuccinimide (0.1202 g, 1.044 mmol) and N,N-Dicyclohexylcarbodiimide (0.2155 g, 1.044 mmol) were dissolved in DMF (2.0 mL) and added to the reaction mixture under a N_2_ atmosphere, with the aid of additional DMF (1.0 mL).

The reaction mixture was stirred at room temperature for 18 h. The resultant suspension was filtered under vacuum to remove dicyclohexyl urea then the solution re-precipitated into rapidly stirring diethyl ether (200 mL), the ether was decanted off and the solid washed with ether (2 × 20 mL) then dried *in vacuo* at 35 °C for 18 h to give 0.5910 g (75 %) of a buff-coloured solid.

This HB-PNIPAM with NHS end groups (0.500 g) was dissolved in distilled water (25 mL) under ice (0–1 °C) (dissolution time ≤ 30 min). Vancomycin.HCl (0.1500 g, 0.101 mmol) was dissolved in water (10.0 mL) then 0.1 M sodium phosphate buffer, pH 7.5 (10.0 mL) was added. The fully mixed solution (20.0 mL) was then added to the previously prepared polymer solution (25 mL) under ice (0–1 °C) with gentle agitation (due to frothy solution formed). The pH was raised to 9.0 < *pH* ≤ 9.5 with 1M NaOH. The solution was stored under refrigeration (4 °C) and after ca. 1h the *pH* monitored to ensure 9.0 < pH ≤ 9.5. The aqueous solution was stored at 4 °C for 18 h (no agitation) then the solution (ca. 30 mL) was directly ultrafiltered with distilled water (3 × 300 mL) to give ca. 30 mL of a concentrated polymer solution. The solution was finally freeze-dried (18 h) to give HB-PNIPAM-Van-A, 0.3738 g (55%); HB-PNIPAM-B, 0.5742 g (82%); HB-PNIPAM-C, 0.4240 g (60%) of white, fluffy solids.
